# Use of Simulation Based Training to Enhance Cardiac Auscultation Proficiency

**DOI:** 10.15694/mep.2020.000258.1

**Published:** 2020-11-18

**Authors:** Matthew Lavoie, Bernard Roth, Jeffrey Kunz

**Affiliations:** 1 Madigan Army Medical Center

**Keywords:** Auscultation, Simulation, Murmur, Education

## Abstract

This article was migrated. The article was marked as recommended.

Introduction:

Cardiopulmonary auscultation is an important skill for medical professionals with deficiencies being well documented with broad implications on healthcare. Providing ideal bedside auscultatory teaching presents many difficulties. Simulation has been used as a means to improve cardiac auscultatory training and circumvent some of these limitations.

Methods:

We studied the use of Harvey © simulation in teaching cardiac murmurs and whether there was improvement in short term knowledge, as well as testing long term knowledge retention, as measured through a standardized test. From 2014-2019, 124 medical students in their 2 ^nd^ and 3 ^rd^ year of school (during the clinical portion of medical school curriculum) rotating through an Internal Medicine rotation completed a 2 hour training course on cardiac auscultation using Harvey © to identify six common cardiac murmurs. The session contained a pretest, didactic session, and posttest.

Results:

124 students participated in the auscultatory training session. Of them, 42 (34%) underwent the session a second time at an average of 1.29 months from their first session. There was statistically significant improvement between tests. Notably, the most often missed murmurs were mitral stenosis and benign (innocent) flow murmur.

Discussion/Conclusions:

As shown in our study, simulation based cardiac auscultatory education is feasible and likely beneficial in medical education as it can be delivered to a large group of trainees and overcomes the challenges of bedside teaching.

## Introduction

Being able to hear, interpret, and communicate findings on cardiopulmonary auscultation is an important skill for medical professionals. The stethoscope is a valuable and cost effective tool that can aid in evaluation of valvular heart disease ( Roldan *et al.*, 1996), dyspnea, and congestive heart failure ( Gillespie *et al.*, 1997). Deficiencies in auscultatory skill throughout medical training have been described. This has implications on medical decision making, diagnosis, patient safety, cost effective care, and continuing medical education ( Woywodt *et al*., 2004, Mangione and Nieman, 1997, Mangione *et al.*, 1993). Historically, cardiac examination skills in medical traniees is poor and proficiency has been found to potentially decline with more years of training past 3 ^rd^ year of medical school, with a lack of cardiac skills being documented in medical students as well as clinicans ( Mangione and Nieman, 1997) ( Mangione *et al.*, 1993) ( Peacock *et al.*, 2006) ( Barrett *et al.*, 2004) ( Lam *et al.*, 2005) ( Perlini *et al.*, 2014) ( Vukanovic-Criley *et al.*, 2006, Mangione, 2001, March *et al.*, 2005).

Indeed, there are many contributors to this lack of auscultation proficiency. There has been a decreased incidence of significant valvular pathlogy with contemporary medicine. In addition, congenital anomalies such as VSD are identified much earlier than once before. This leads to a reduced number of patients with cardiac murmurs and decreased educational opportunity to learn important pathology ( Birdane *et al.*, 2012). Finally, providing ideal bedside auscultatory teaching presents many difficulties to include time constraints on students and experts, patient privacy and difficulty in finding a patient with a significant murmur who is agreeable to bedside teaching.. Consequently, simulation has been used as a means to improve cardiac auscultatory training and circumvent some of these limitations.

Many means of auscultory education exist to include audio, web based, ausucltation task trainers, high fidelity mannequins, recording sthetoscopes, auscultation sound playback devices and sthethescopes. Mannequin simulators, such as Harvey, offer a more realistic patient encounter than audio recordings alone ( Ward and Wattier, 2011).

Curricula have been developed to train and help learners retain cardiac auscultation skills. Several studies, including a metanalysis, have shown the benefit of cardiac ausculatory training in learning and retaining ausculatory skills - from medical students to residents ( Perlini *et al.*, 2014) ( Ewy *et al.*, 1987) ( Spatz *et al.*, 2011) ( Fraser *et al.*, 2011, Butter *et al.*, 2010) ( McKinney *et al.*, 2013). Unfortunately, cardiac auscultation training can be highly variable or even not routinely available during medical school training. Appropriate diagnostic auscultation is particularly important in military medical settings where sophisticated medical diagnostic technology may not be available.

We studied the use of Harvey © simulation in teaching cardiac murmurs and whether there were improvements in short and long term knowledge retention, as measured through a standardized test. This study was conducted over the course of 4 years.

## Methods

Madigan Army Medical Center is a large regional military medical treatment facility that has had medical students rotating through several specialties for many years. Prior to 2014, no formal cardiac auscultation training existed for students rotating through the required Internal Medicine medical school inpatient and outpatient rotations. Thus, such a curriculum was developed using the Harvey© simulator.

The Harvey© simulator was the earliest high fidelity technology for cardiac auscultation and was developed in 1968 by Dr. Michael Gordon. It is a full torso mannequin fixed in the supine position to a table. Specific pathology can be set by changing controls on the mannequin console. The Harvey simulator works through transmission of infrared signals from a transmitter to a master stethoscope as a learner places the stethoscope on the mannequin’s chest. When the stethoscope is placed on specific areas of the chest preprogrammed sound playback is produced. The Harvey simulator is meant to give the impression of listening to a real patient. For example: murmurs are different when listening in different areas, peripheral pulses can be palpated during auscultation, and jugular vein pulsations can be observed. This simulation technology has been validated in a number of studies ( Ewy *et al.*, 1987) ( Woolliscroft *et al.*, 1987).

From 2014-2019, 124 medical students in their 2 ^nd^ and 3 ^rd^ year of school (during the clinical portion of medical school curriculum) rotating through an Internal Medicine rotation completed a 2 hour training course on cardiac auscultation using Harvey © to identify six common cardiac murmurs: aortic stenosis, aortic regurgitation, mitral stenosis, mitral regurgitation, hypertrophic cardiomyopathy, and benign (innocent) flow murmurs. A pretest was first performed in which students were given a standardized clinical vignette, listened to one of six heart murmurs and then were asked to identify the murmur. Next, a formal 2 hour didactic session was held which consisted of a review of the cardiac physical exam including vital signs, observation, palpation, and auscultation. The auscultory education included teaching how to categorize and grade heart murmurs as well as recognize normal and abnormal heart sounds. Each of the previous six murmurs were reviewed. After the didactic session a post test was conducted.

The posttest consisted of the same six murmurs, but tested in a different order and with a different standardized clinical scenario than the pretest. Results were tabulated to show an overall percentage of correctly identified murmurs, as well as per individual murmur. Possible scores ranged from 0 (none correct) to 6 (all correct). Subsequent analysis was performed for each individual murmur. Additionally, 42 students went through a second internal medicine rotation at Madigan Army Medical Center and thus underwent the training a second time. This occurred either 1 or 2 months later for the students. Their pre and post test results were also collected.

The students pre and post test results were recorded and analyzed. The null hypothesis was that the mean test scores of the pretest and posttest would not differ. Test results were compared using simple t tests. Data were analyzed using Microsoft excel software, and a p<0.05 was considered statistically significant. This quality improvement project was reviewed and approved by the Madigan Army Medical Center Human Research Protections Office.

## Results

**Figure 1.**
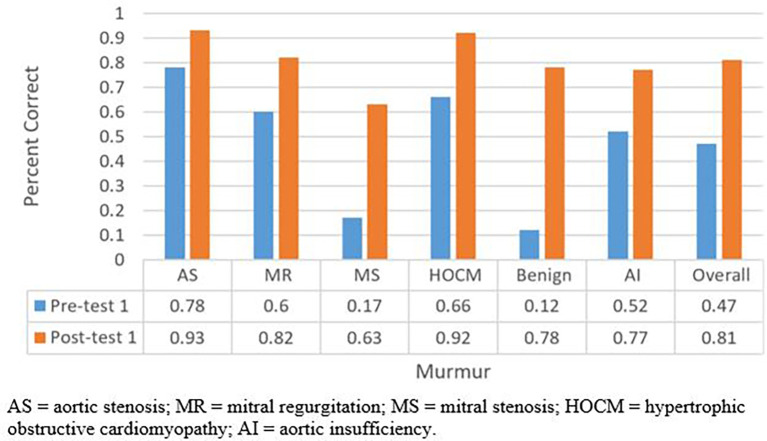
Total percentage of questions answered correctly for pre-test 1 and post-test 1.

**Figure 2.**
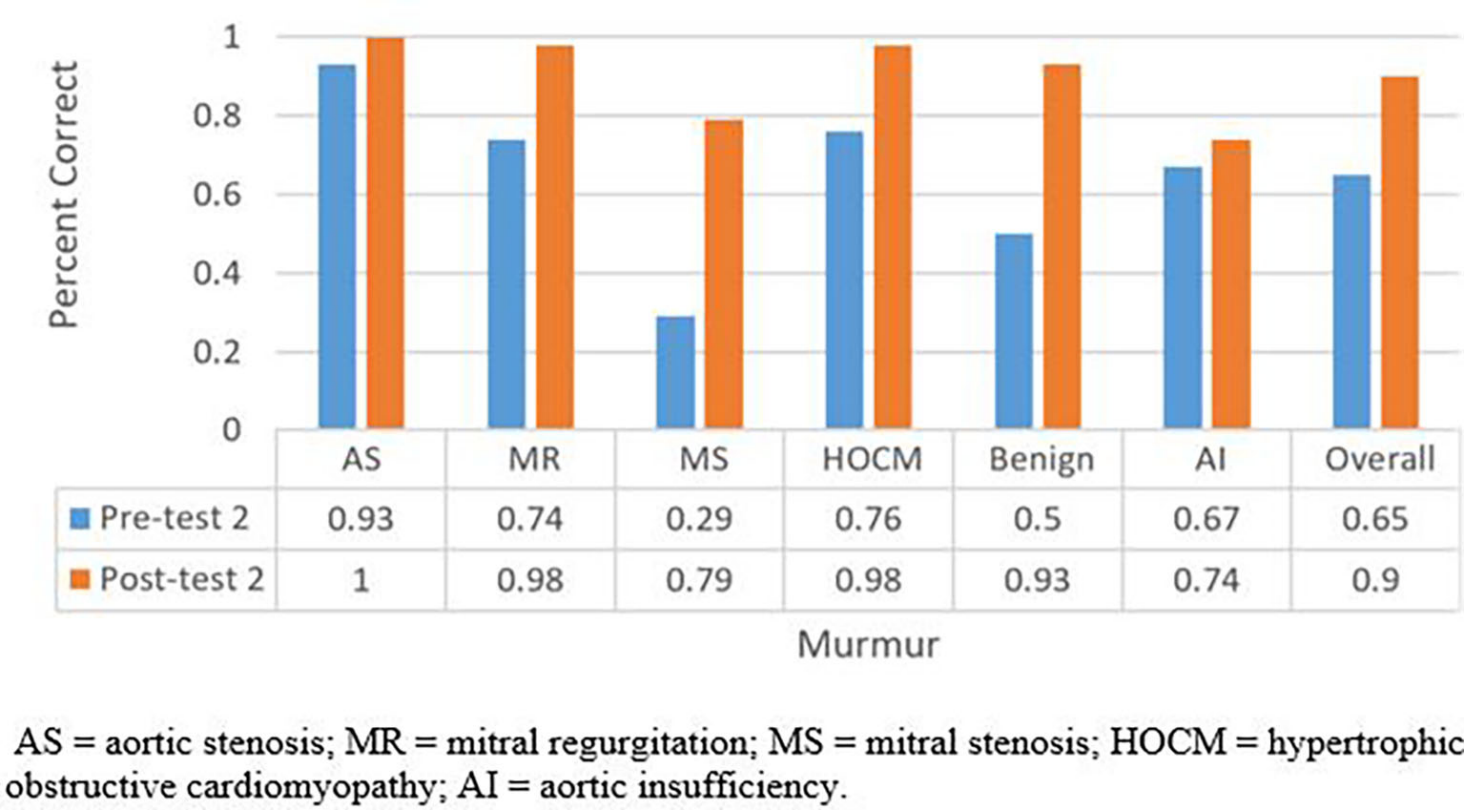
Total percentage of questions answered correctly for pre-test 2 and post-test 2.

**Figure 3.**
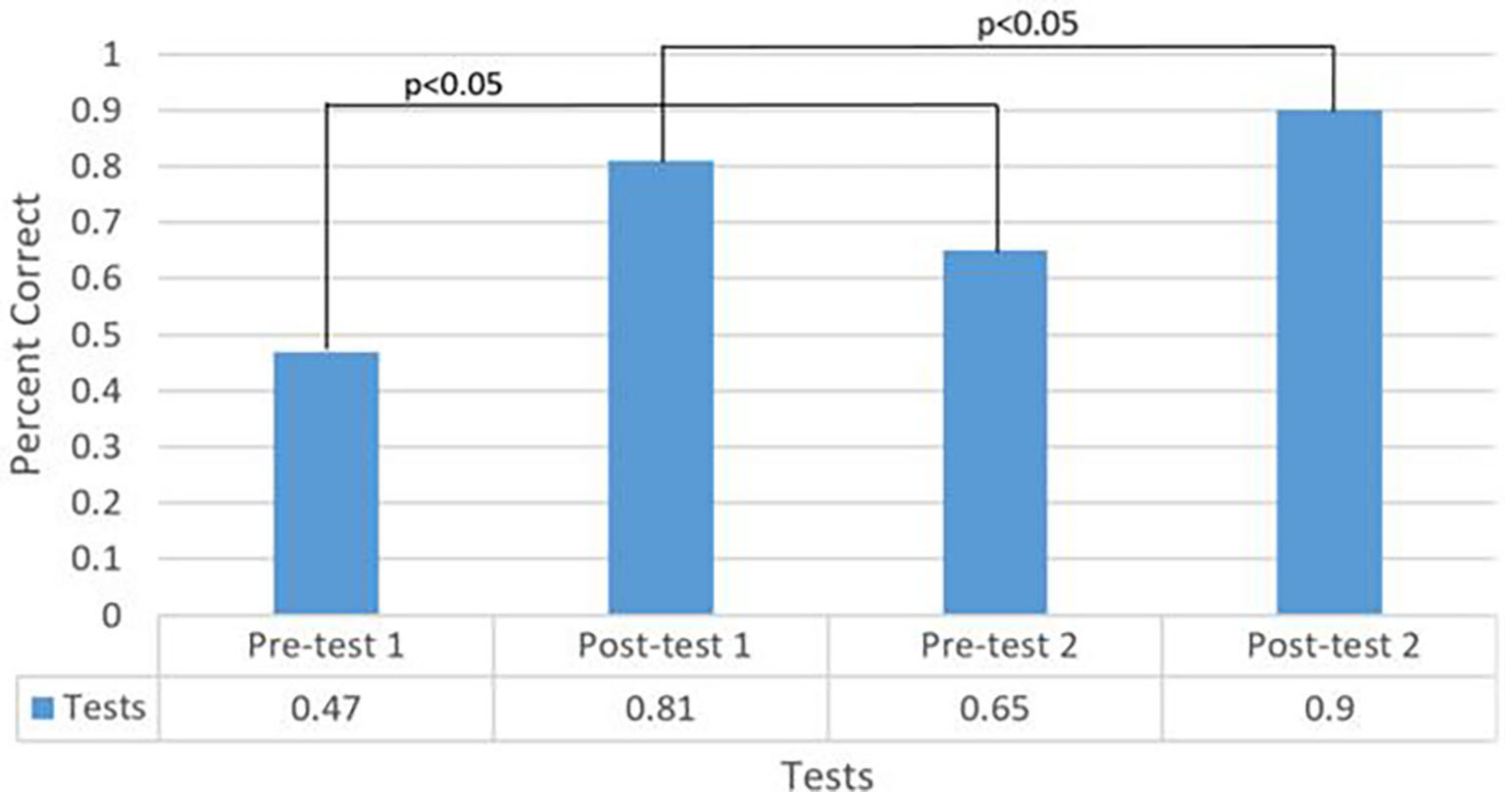
Total percentage of questions answered correctly in all tests.

124 students participated in the auscultatory training session. Of them, 42 (34%) underwent the session a second time at an average of 1.29 months from their first session. The average scores of pretest 1, posttest 1, pretest 2, and posttest 2 were 47%, 81%, 65%, and 90 % respectively. Students improved from pretest 1 to posttest 1 (47% to 81%, p<0.001; Figure 1), pretest 2 to posttest 2 (65% to 90%, P<0.001; Figure 2); pretest 1 to pretest 2 (47% to 65%, p<0.001; Figure 3), and posttest 1 to posttest 2 (81% to 90%, p=0.001; Figure 3). Notably, the most often missed murmurs were mitral stenosis and benign (innocent) flow murmur.

Pre-test 1/Post-test 1

Pre-test 2/ Post-test 2

All Tests

## Discussion

The cardiovascular physical exam is a noninvasive, cost-effective tool useful in the preliminary diagnosis of valvular heart disease that requires further evaluation. Mastering the cardiac physical exam outside of simulation based training would require examining patients with a wide variety of cardiac conditions and encountering these diagnoses on a repetitive basis which is not feasible.

Simulation based medical education can allow repetitive and planned exposure to key ausculatory abnormalities without the constraints previously discussed. Simulation based physical examination training can likely be implemented as a stand alone intervention and does not necessarily have to be integrated into a specific curriculum ( McKinney *et al.*, 2013). Unfortunately, based on a recent survery of UK medical schools, cardiac simulation is not being used as a tool for repetitive practice, but mainly as a means for introduction to heart murmurs. Of note, the impact of such teaching on clinical examination has generally not been measured ( Owen and Wong, 2015).

Studies have used cardiac simulation to teach learners from medical student to attending, with promising results ( McKinney *et al.*, 2013). This type of intervention is feasible for medical school and residency programs as it can be delivered to a large group of trainees and overcomes the challenges of bedside teaching. Presumably, this translates to improvement in cardiac ausculation in actual patients.

One recent analysis of embedded multimedia simulations of heart sounds on the United States Medical Licensing Exam found that medical students appear to have become more adept at interpreting auscultation findings in recent years (2012 compared to 2007). The authors suggested this improvement may be due to technologic advances facilitating reproduction of heart sounds allowing for production of educational cases. Another consideration was that examinees may be receiving more formal training in cardiac auscultation from their medical schools ( Short *et al.*, 2018).

As shown in our study, simulation based cardiac auscultatory education is feasible and likely beneficial in medical education as it can be delivered to a large group of trainees and overcomes the challenges of bedside teaching. This training also has increased potential benefit in military medical trainees that may find themselves in resource limited settings.

Future research could focus on assessing cardiac diagnostic skill in real clinical practice, as compared to cardiac simulation. Additional studies may also explore longitudinal auscultation skills throughout a physician’s career. There are several limitations to this study. Our study lacked a control group that could be compared to the efficacy of simulation based cardiac training. It is also not clear if the observed improvement in murmur identification will translate to actual patients since all testing was done on the simulator. In addition, the study was conducted in one center, with a relatively small sample size, and was limited to available medical trainees at this institution.

## Conclusion

As shown in our study, simulation based cardiac auscultatory education is feasible and likely beneficial in medical education as it can be delivered to a large group of trainees and overcomes the challenges of bedside teaching. This training also has increased potential benefit in military or rural medical trainees that may find themselves in resource limited settings.

## Take Home Messages

• Cardiopulmonary auscultation is an important skill for medical professionals and has broad implications on healthcare. Despite this, deficiencies are well documented.
• Particularly difficult to diagnose murmurs appear to include mitral stenosis, aortic insufficiency, and benign (innocent) flow murmur.
• Simulation based cardiac auscultatory education is feasible and likely beneficial in medical education as it can be delivered to a large group of trainees and overcomes the challenges of bedside teaching.

## Notes On Contributors

**Dr. Matthew Lavoie**, MD is an internal medicine resident at Madigan Army Medical Center, United States of America.

**Dr. Bernard Roth**, MD is a board certified pulmonary and critical care physician at Madigan Army Medical Center, United States of America.

**Dr. Jeffrey Kunz**, MD is a board certified interventional cardiologist at Madigan Army Medical Center, United States of America.
