# Supplementary material for: Use of Simulation Based Training to Enhance Cardiac Auscultation Proficiency
Source: MedEdPublish (2016). 2020 Nov 18;9:258. [Version 1] doi: 10.15694/mep.2020.000258.1 (PMC10697582; doi:10.15694/mep.2020.000258.1)
Supplement: Peer review history

# Reviewer report: Reviewer response for version 1

**DOI:** 10.21956/mep.20173.r31184

**Published:** 2021-04-13

**Authors:** Julie Hunt

**Affiliations:** 1 Lincoln Memorial University

This review has been migrated. The reviewer awarded 4 stars out of 5 This study describes a simulation-based training study with a pre-test/post-test design which repeated 1-2 months later. The study provides evidence that students learned from the intervention, lost some knowledge over the 1-2 month period, but retained some of what they learned. This is promising for Harvey simulator training in cardiac auscultation.What the study does not contain is a validation of the simulator. The authors do not include a reference to suggest whether that has been performed previously, or a statement about whether that should be performed subsequently—but surely one statement or the other is appropriate to include to ensure that the simulator is valid for teaching and assessing the skill. The authors do mention in the discussion that there is a presumption that skills demonstrated on the mannikin translate into skills demonstrated on live patients, and this is a presumption that should be investigated by future research.I would add as a limitation of this research that the study did not gather any student feedback on the simulator, nor any expert opinions of the simulator. This feedback may have offered explanations for why the mitral stenosis and innocent flow murmurs were most challenging to hear, or have generated feedback for improvement of the educational experience.Overall I think this study is a valuable start to investigating Harvey’s use for teaching auscultation. I would like to see additional testing to validate and support the simulator’s use for this purpose.

Reviewer Expertise:

NA

No decision status is available

# Reviewer report: Reviewer response for version 1

**DOI:** 10.21956/mep.20173.r31183

**Published:** 2021-03-06

**Authors:** Ken Masters

**Affiliations:** 1 Sultan Qaboos University

This review has been migrated. The reviewer awarded 4 stars out of 5 An interesting paper dealing with the use of simulation-based training to enhance cardiac auscultation proficiency.The introduction clearly identifies the main problems with teaching the topic: there is a great need for the skills, but finding suitable patients on which to learn and practice is difficult. A simulation device has the potential to help solve the problem. The experimental design follows a standard pre-test, intervention and post-test, and the results are compared. Some small issues:• The results are clearly laid out, although some analysis would have been useful (this may be personal preference).• If there are no copyright or other issues, it would be useful if the contents (or, at least, a detailed course outline) of the intervention could be given. This would assist readers in their implementation of such an intervention – and this would be the real value of the paper: not merely telling people that it CAN be done, but giving them greater insight into HOW it can be done.• The mean percentages should be given with one decimal place.• It would be useful if the Discussion could begin with a brief synopsis of what was performed.Although this is a small and localised study, it is a useful contribution to the value of using simulation-based interventions in medical teaching.

Reviewer Expertise:

NA

No decision status is available

# Reviewer report: Reviewer response for version 1

**DOI:** 10.21956/mep.20173.r31182

**Published:** 2021-02-28

**Authors:** Haipeng Xiao

**Affiliations:** 1 The First Affiliated Hospital, Sun Yat-sen University

This review has been migrated. The reviewer awarded 3 stars out of 5 This is an exploratory study that looked into the effect of simulation training on cardiac auscultatory skills among a convenient sample of second-year and third-year medical students and found that such an educational approach was both practically feasible and potentially beneficial. Besides what was mentioned by the authors in the limitation section, I believe this piece of research could be improved upon in several ways, when resources permit: (1) adding a control arm (as already mentioned in the manuscript); (2) on top of having a control arm, try to use the 4-group design to account for the effect of pre-test; (3) collect quantitative data on course satisfaction as well as qualitative data on learning experiences among students, to further explore the educational impact of this intervention; (4) assess students' performance in real-world clinical practice (for "transfer of learning"). In any case, this work is worth reading for those working in the area of simulation.

Reviewer Expertise:

NA

No decision status is available
